# Recurrent neural network-based volumetric fluorescence microscopy

**DOI:** 10.1038/s41377-021-00506-9

**Published:** 2021-03-23

**Authors:** Luzhe Huang, Hanlong Chen, Yilin Luo, Yair Rivenson, Aydogan Ozcan

**Affiliations:** 1 Electrical and Computer Engineering Department, University of California, Los Angeles, CA 90095 USA; 2 Bioengineering Department, University of California, Los Angeles, CA 90095 USA; 3 California Nano Systems Institute (CNSI), University of California, Los Angeles, CA 90095 USA; 4 David Geffen School of Medicine, University of California, Los Angeles, CA 90095 USA

**Keywords:** Imaging and sensing, Microscopy

## Abstract

Volumetric imaging of samples using fluorescence microscopy plays an important role in various fields including physical, medical and life sciences. Here we report a deep learning-based volumetric image inference framework that uses 2D images that are sparsely captured by a standard wide-field fluorescence microscope at arbitrary axial positions within the sample volume. Through a recurrent convolutional neural network, which we term as Recurrent-MZ, 2D fluorescence information from a few axial planes within the sample is explicitly incorporated to digitally reconstruct the sample volume over an extended depth-of-field. Using experiments on *C. elegans* and nanobead samples, Recurrent-MZ is demonstrated to significantly increase the depth-of-field of a 63×/1.4NA objective lens, also providing a 30-fold reduction in the number of axial scans required to image the same sample volume. We further illustrated the generalization of this recurrent network for 3D imaging by showing its resilience to varying imaging conditions, including e.g., different sequences of input images, covering various axial permutations and unknown axial positioning errors. We also demonstrated wide-field to confocal cross-modality image transformations using Recurrent-MZ framework and performed 3D image reconstruction of a sample using a few wide-field 2D fluorescence images as input, matching confocal microscopy images of the same sample volume. Recurrent-MZ demonstrates the first application of recurrent neural networks in microscopic image reconstruction and provides a flexible and rapid volumetric imaging framework, overcoming the limitations of current 3D scanning microscopy tools.

## Introduction

High-throughput imaging of 3D samples is of significant importance for numerous fields. Volumetric imaging is usually achieved through optical sectioning of samples using various microscopy techniques. Generally, optical sectioning can be categorized based on its dimension of sectioning: (i) 0-dimensional point-wise sectioning, including e.g., confocal^1^, two-photon^2^ and three-photon^3^ laser scanning microscopy, and time-domain optical coherence tomography (TD-OCT)^4^; (ii) 1-dimensional line-wise sectioning, including e.g., spectral domain OCT^5,6^, (iii) 2-dimensional plane-wise sectioning, including e.g., wide-field and light-sheet^7^ fluorescence microscopy. In all of these modalities, serial scanning of the sample volume is required, which limits the imaging speed and throughput, reducing the temporal resolution, also introducing potential photobleaching on the sample. Different imaging methods have been proposed to improve the throughput of scanning-based 3D microscopy techniques, such as multifocal imaging^8–13^, light-field microscopy^14,15^, microscopy with engineered point spread functions (PSFs)^16–18^ and compressive sensing^19–21^. Nevertheless, these solutions introduce trade-offs, either by complicating the microscope system design, compromising the image quality and/or resolution or prolonging the image post-processing time. In addition to these, iterative algorithms that aim to solve the inverse 3D imaging problem from a lower dimensional projection of the volumetric image data, such as the fast iterative shrinkage and thresholding algorithm (FISTA)^22^ and alternating direction method of multiplier (ADMM)^23^ are relatively time-consuming and unstable, and further require user-defined regularization of the optimization process as well as an accurate forward model of the imaging system. Some of these limitations and performance trade-offs have partially restricted the wide-scale applicability of these computational methods for 3D microscopy.

In recent years, emerging deep learning-based approaches have enabled a new set of powerful tools to solve various inverse problems in microscopy^24,25^, including e.g., super-resolution imaging^26,27^, virtual labeling of specimen^28–32^, holographic imaging^33,34^, Fourier ptychography microscopy^35^, single-shot autofocusing^36,37^, three-dimensional image propagation^38^, among many others^39^. Benefiting from the recent advances in deep learning, these methods require minimal modification to the underlying microscopy hardware, and result in enhanced imaging performance in comparison to conventional image reconstruction and post-processing algorithms.

The majority of these neural networks applied in microscopic imaging were designed to perform inference using a single 2D input image. An alternative method to adapt a deep network’s inference ability to utilize information that is encoded over volumetric inputs (instead of a single 2D input image) is to utilize 3D convolution kernels. However, this approach requires a significant number of additional trainable parameters and is therefore more susceptible to overfitting. Moreover, simply applying 3D convolution kernels and representing the input data as a sequence of 2D images would constrain the input sampling grid and introduce practical challenges. As an alternative to 3D convolution kernels, recurrent neural networks (RNNs) were originally designed for sequential temporal inputs, and have been successfully applied in various tasks in computer vision^40–42^.

**Fig. 1:**
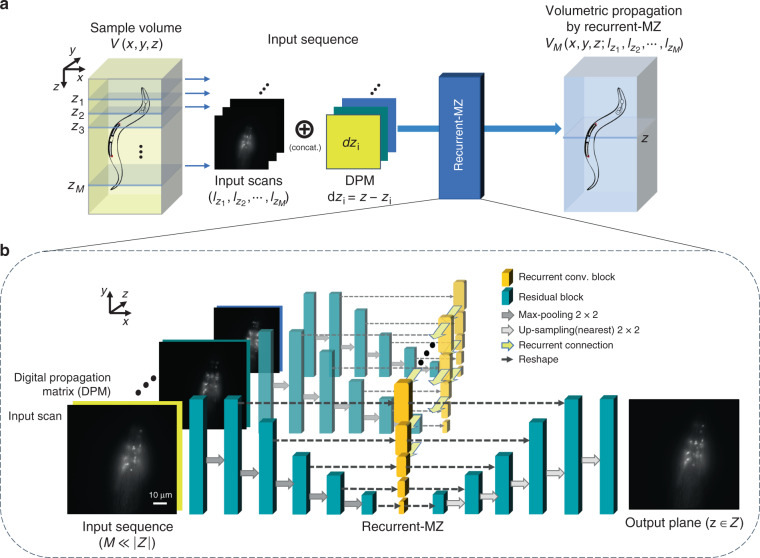
Volumetric imaging through Recurrent-MZ. **a** Recurrent-MZ volumetric imaging framework. *M* is the number of input scans (2D images), and each input scan is paired with its corresponding DPM (Digital Propagation Matrix). **b** Recurrent-MZ network structure. The network and training details are elucidated in the Materials and Methods section. *Z* is the set of all the discretized axial positions within the target sample volume, composed of |*Z*| unique axial planes. Typically, *M* = 2 or 3 and |*Z*| ≫ *M*

Here, we introduce the first RNN-based volumetric microscopy framework, which is also the first application of RNNs in microscopic image reconstruction; we termed this framework as Recurrent-MZ. Recurrent-MZ permits the digital reconstruction of a sample volume over an extended depth-of-field (DOF) using a few different 2D images of the sample as inputs to a trained RNN (see Fig. 1a). The input 2D images are sparsely sampled at arbitrary axial positions within the sample volume and the convolutional recurrent neural network (Recurrent-MZ) takes these 2D microscopy images as its input, along with a set of digital propagation matrices (DPMs) which indicate the *relative distances* (*dz*) to the desired output plane(s). Information from the input images is separately extracted using sequential convolution blocks at different scales, and then the recurrent block aggregates all these features from the previous scans/images, allowing flexibility in terms of the length of the input image sequence as well as the axial positions of these input images, which do not need to be regularly spaced or sampled; in fact, the input 2D images can even be randomly permuted.

We demonstrate the efficacy of the Recurrent-MZ using multiple fluorescent specimens. First, we demonstrate Recurrent-MZ inference for 3D imaging of *C. elegans* samples, and then quantify its performance using fluorescence nanobeads. Our results demonstrate that Recurrent-MZ significantly increases the depth-of-field of a 63×/1.4NA objective lens, providing a 30-fold reduction in the number of axial scans required to image a sample volume. Furthermore, we demonstrate the robustness of this framework and its inference to axial permutations of the input images as well to uncontrolled errors and noise terms in the axial positioning of different input image scans. Finally, we report wide-field to confocal cross-modality image transformation using the Recurrent-MZ framework, which takes in e.g., three wide-field 2D fluorescence images of a sample as input in order to reconstruct a 3D image stack, matching confocal microscopy images of the same sample; we refer to this cross-modality image transformation network as Recurrent-MZ+.

## Results

We formulate the target sample volume *V*(*x*,*y*,*z*) as a random field on the set of all discretized axial positions *Z*, i.e., $I_z \in {\mathbb {R}}^{m \times n},z \in Z$, where *x*,*y* are pixel indices on the lateral plane, *m*, *n* are the lateral dimensions of the image, and *z* is a certain axial position in *Z*. The distribution of such random fields is defined by the 3D distribution of the sample of interest, the PSF of the microscopy system, the aberrations and random noise terms present in the image acquisition system. Recurrent-MZ takes in a set of *M* 2D axial images, i.e., $\left\{ {I_{z_1},I_{z_2}, \cdots ,I_{z_M}} \right\},\,1\, < \,M \ll |Z|$, where |*Z*| is the cardinality of *Z*, defining the number of unique axial planes in the target sample. The output inference of Recurrent-MZ estimates (i.e., reconstructs) the volume of the sample and will be denoted as $V_M(x,y,z;\,I_{z_1},I_{z_2}, \cdots ,I_{z_M})$. Starting with the next sub-section we summarize Recurrent-MZ inference results using different fluorescent samples.

### Recurrent-MZ based volumetric imaging of C. elegans samples

**Fig. 2:**
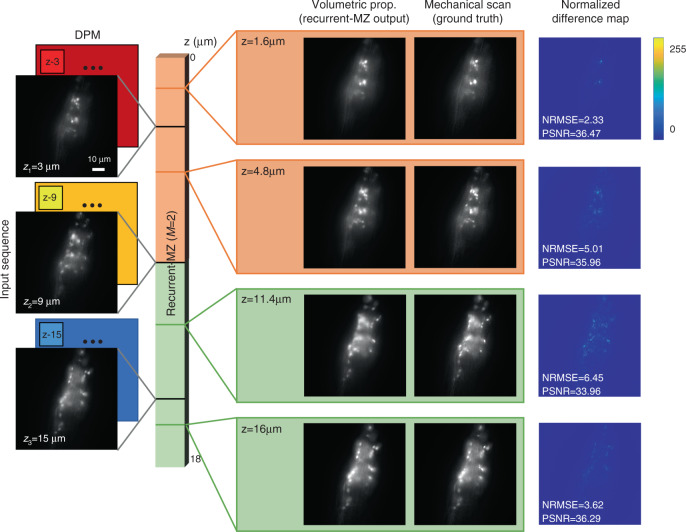
Volumetric imaging of *C. elegans* from sparse wide-field scans using Recurrent-MZ. The DPMs in the input sequence are used to define an arbitrary axial position (*z*) within the sample volume. In this implementation, Recurrent-MZ takes in 2 input scans (*M* = 2) to infer the image of an output plane, as indicated by the color of each output box. See Video S1 to compare the reconstructed sample volume inferred by Recurrent-MZ against the ground truth, |Z| = 91 images captured with an axial step size of 0.2 μm

A Recurrent-MZ network was trained and validated using *C. elegans* samples, and then blindly tested on new specimens that were *not* part of the training/validation dataset. This trained Recurrent-MZ was used to reconstruct *C. elegans* samples with high fidelity over an extended axial range of 18 μm based on three 2D input images that were captured with an axial spacing of Δ*z* = 6 μm; these three 2D images were fed into Recurrent-MZ in groups of two, i.e., *M* = 2 (Fig. 2). The comparison images of the same sample volume were obtained by scanning a wide-field fluorescence microscope with a 63×/1.4NA objective lens and capturing |*Z*| = 91 images with an axial spacing of Δ*z* = 0.2 μm (see the Materials and Methods section). The inference performance of Recurrent-MZ is both qualitatively and quantitatively demonstrated in Fig. 2 and Video S1. Even in the middle of two adjacent input images (see the *z* = 11.4 μm row of Fig. 2), Recurrent-MZ is able to output images with a very good match to the ground truth image, achieving a normalized root mean square error (NRMSE) of 6.45 and a peak signal-to-noise ratio (PSNR) of 33.96. As also highlighted in Video S1, Recurrent-MZ is able to significantly extend the axial range of the reconstructed images using only three 2D input scans, each captured with a 1.4NA objective lens that has a depth-of-field of 0.4 μm. In addition to these, Supplementary Note 1 and Fig. S1 also compare the output images of Recurrent-MZ with the results of various interpolation algorithms, further demonstrating the advantages of Recurrent-MZ framework for volumetric imaging.

It is worth noting that although Recurrent-MZ presented in Fig. 2 was trained with 2 input images (i.e., *M* = 2), it still can be fed with *M* ≥ 3 input images thanks to its recurrent scheme. *Regardless of the choice of M, all Recurrent-MZ networks have the same number of parameters, where the only difference is the additional time that is required during the training and inference phases*; for example the inference time of Recurrent-MZ with *M* = 2 and *M* = 3 for a single output plane (1024 × 1024 pixels) is 0.18 s and 0.28 s, respectively. In practice, using a larger *M* yields a better performance in terms of the reconstruction fidelity (see e.g., Fig. S2a), at the cost of a trade-off of imaging throughput and computation time. The detailed discussion about this trade-off is provided in the Discussion section.

### Recurrent-MZ based volumetric imaging of fluorescence nanobeads

**Fig. 3:**
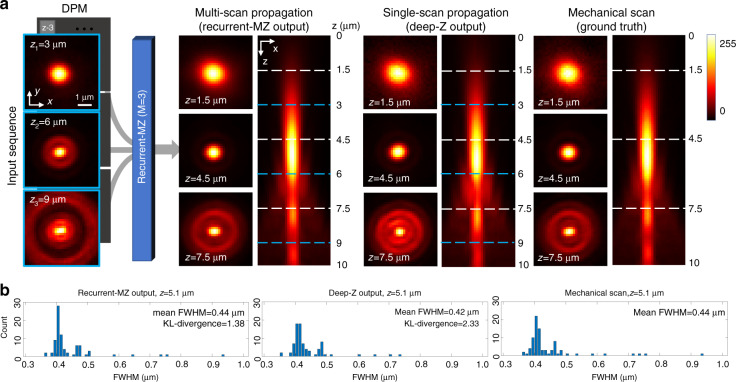
The performance of Recurrent-MZ using fluorescence nanobeads. **a** Volumetric imaging using Recurrent-MZ (*M* = 3) and Deep-Z on 50 nm fluorescence nanobeads. There are 3 input images for Recurrent-MZ (*M* = 3) and to provide a fair comparison, Deep-Z always takes in the nearest input image among these 3 inputs to infer another axial plane. The PSFs generated by Recurrent-MZ, Deep-Z and mechanical scanning (Δ*z* = 0.1 μm) are shown for comparison. **b** FWHM histograms for 88 individual isolated fluorescence nanobeads at *z* = 5.1 μm, measured from mechanical scanning (101 axial images), Deep-Z reconstruction and Recurrent-MZ reconstruction (*M* = 3). Also see Video S2

Next, we demonstrated the performance of Recurrent-MZ using 50 nm fluorescence nanobeads. These nanobead samples were imaged through the TxRed channel using a 63×/1.4NA objective lens (see the Materials and Methods section). The Recurrent-MZ model was trained on a dataset with *M* = 3 input images, where the axial spacing between the adjacent planes was Δ*z* = 3 μm. The ground truth images of the sample volume were captured by mechanical scanning over an axial range of 10 μm, i.e., |*Z*| = 101 images with Δ*z* = 0.1 μm were obtained. Figure 3 shows both the side views and the cross-sections of the sample volume reconstructed by Recurrent-MZ (*M* = 3), compared against the |*Z*| = 101 images captured through the mechanical scanning of the same sample. The first column of Fig. 3a presents the *M* = 3 input images and their corresponding axial positions, which are also indicated by the blue dashed lines. Through the quantitative histogram comparison shown in Fig. 3b, we see that the reconstructed volume by Recurrent-MZ matches the ground truth volume with high fidelity. For example, the full width at half maximum (FWHM) distribution of individual nanobeads inferred by Recurrent-MZ (mean FWHM = 0.4401 μm) matches the results of the ground truth (mean FWHM = 0.4428 μm) very well. We also showed the similarity of the ground truth histogram with that of the Recurrent-MZ output by calculating the Kullback–Leibler (KL) divergence, which is a distance measure between two distributions; the resulting KL divergence of 1.3373 further validates the high fidelity of Recurrent-MZ reconstruction when compared to the ground truth, acquired through |*Z*| = 101 images captured via mechanical scanning of the sample with Δ*z* = 0.1 μm.

Figure 3 also reports the comparison of Recurrent-MZ inference results with respect to another fluorescence image propagation network termed Deep-Z^38^. Deep-Z is designed for taking a *single* 2D image as input, and therefore there is an inherent trade-off between the propagation quality and the axial refocusing range (from a given focal plane), which ultimately limits the effective volumetric space-bandwidth-product (SBP) that can be achieved using Deep-Z. In this comparison between Recurrent-MZ and Deep-Z (Fig. 3), the nearest input image is used for Deep-Z based propagation; in other words, three non-overlapping volumes are separately inferred using Deep-Z from the input scans at *z* = 3, 6 and 9 μm, respectively (this provides a fair comparison against Recurrent-MZ with *M* = 3 input images). As illustrated in Fig. 3b, Deep-Z inference resulted in a mean FWHM of 0.4185 μm and a KL divergence of 2.3334, which illustrate the inferiority of single-image-based volumetric propagation, when compared to the results of Recurrent-MZ. The same conclusion regarding the performance comparison of Recurrent-MZ and Deep-Z inference is further supported using the *C. elegans* imaging data reported in Fig. 2 (Recurrent-MZ) and in Fig. S3 (Deep-Z). For example, Deep-Z inference results in an NRMSE of 8.02 and a PSNR of 32.08, while Recurrent-MZ (*M* = 2) improves the inference accuracy, achieving an NRMSE of 6.45 and a PSNR of 33.96.

### Generalization of Recurrent-MZ to non-uniformly sampled input images

**Fig. 4:**
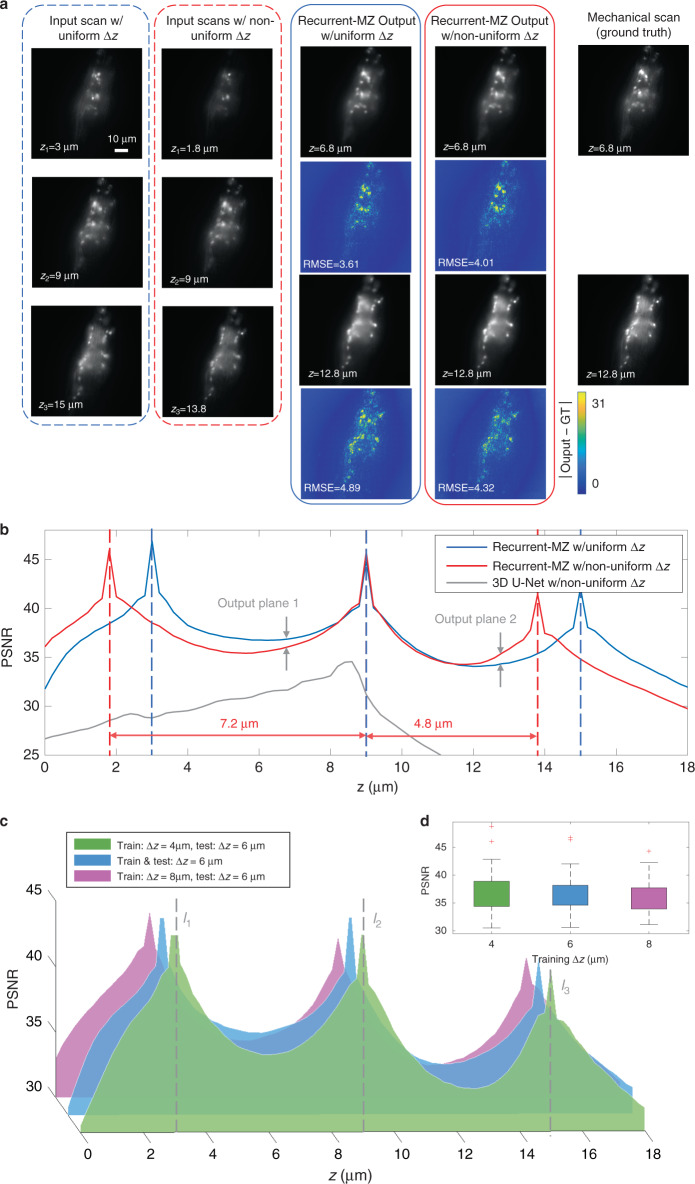
Generalization of Recurrent-MZ to non-uniformly spaced input images. **a** Recurrent-MZ was trained on C. elegans samples with equidistant inputs (*M* = 3, Δ*z* = 6 μm), and blindly tested on both uniformly sampled and non-uniformly sampled input images of new samples. **b** The PSNR values of the output images of Recurrent-MZ with uniformly spaced and non-uniformly spaced input images, as well as the output images of 3D U-Net with non-uniformly spaced input images are all calculated with respect to the ground truth, corresponding image. Blue: Outputs of Recurrent-MZ (*M* = 3) for uniformly spaced inputs, Red: Outputs of Recurrent-MZ (*M* = 3) for non-uniformly spaced inputs, Gray: Outputs of 3D U-Net for non-uniformly spaced inputs (lower PSNR values are omitted). Dashed lines indicate the axial positions of the input 2D images. **c** Influence of hyperparameter Δ*z* on Recurrent-MZ inference performance. We report the PSNR values of the output images of Recurrent-MZ (*M* = 3) models that were trained using different Δ*z* = 4, 6, and 8 μm, but blindly tested on new samples imaged with Δ*z* = 6 μm. The input images are captured at *z* = 3, 6, and 9 μm. **d** The boxplot of the PSNR values of the 3 networks (trained using Δ*z* = 4, 6 and 8 μm)

Next, we demonstrated, through a series of experiments, the generalization performance of Recurrent-MZ on *non-uniformly* sampled input images, in contrast to the training regiment, which only included uniformly spaced inputs. These non-uniformly spaced input image planes were randomly selected from the same testing volume as shown in Fig. 2, with the distance between two adjacent input planes made smaller than the uniform axial spacing used in the training dataset (Δ*z* = 6 μm). Although the Recurrent-MZ was solely trained with *equidistant* input scans, it generalized to successfully perform volumetric image propagation using *non-uniformly sampled* input images. For example, as shown in Fig. 4a, the input images of Recurrent-MZ were randomly selected at (*z*_1_, *z*_2_, *z*_3_) = (3, 7.8, 13.6) μm, respectively, and the output inference at *z* = 6.8 μm and *z* = 12.8 μm very well match the output of Recurrent-MZ that used uniformly sampled inputs acquired at (*z*_1_, *z*_2_, *z*_3_) = (3, 9, 15) μm, respectively. Figure 4b further demonstrates the inference performance of Recurrent-MZ using non-uniformly sampled inputs throughout the specimen volume. The blue (uniform inputs) and the red curves (non-uniform inputs) in Fig. 4b have very similar trends, illustrating the generalization of Recurrent-MZ, despite being only trained with uniformly-sampled input images with a fixed Δ*z*. Figure S3 further presents another successful blind inference of Recurrent-MZ on non-uniformly sampled input images. On the other hand, the gray curve in Fig. 4b (3D U-Net with the same non-uniform inputs) clearly illustrates the generalization failure of a *non-recurrent* convolutional neural network (CNN) on non-uniformly sampled input images.

We further investigated the effect of the hyperparameter Δz on the performance of Recurrent-MZ. For this, three different Recurrent-MZ networks were trained using Δ*z* = 4, 6, and 8 μm, respectively, and then blindly tested on a new input sequence with Δ*z* = 6 μm. Figure 4c, d show the trade-off between the peak performance and the performance consistency over the inference axial range: by decreasing Δ*z*, Recurrent-MZ demonstrates a better peak inference performance, indicating that more accurate propagation has been learned from smaller Δ*z*, whereas the variance of PSNR, corresponding to the performance consistency over a larger axial range, is degraded for smaller Δ*z*.

### Inference stability of Recurrent-MZ

During the acquisition of the input scans, inevitable measurement errors are introduced by e.g., PSF distortions and focus drift^42^, which jeopardize both the precision and accuracy of the axial positioning measurements. Hence, it is necessary to take these effects into consideration and examine the stability of the Recurrent-MZ inference. For this, Recurrent-MZ was tested on the same image test set as in Fig. 2, only this time, independent and identically distributed (i.i.d.) Gaussian noise was injected into the DPM of each input image, mimicking the measurement uncertainty when acquiring the axial scans. The noise was added to the DPM as follows:

$$Z_{i,noised} = Z_i + z_{d,i}\,J,\,i = 1,2, \cdots ,M$$

where *Z*_*i*_ is the DPM (*m* × *n* matrix) of the i-th input image, *z*_*d*,*i*_ ~ *N*(0,*σ*^2^), *i* = 1, 2, ..., *M* and *J* is an all-one *m* × *n* matrix.

**Fig. 5:**
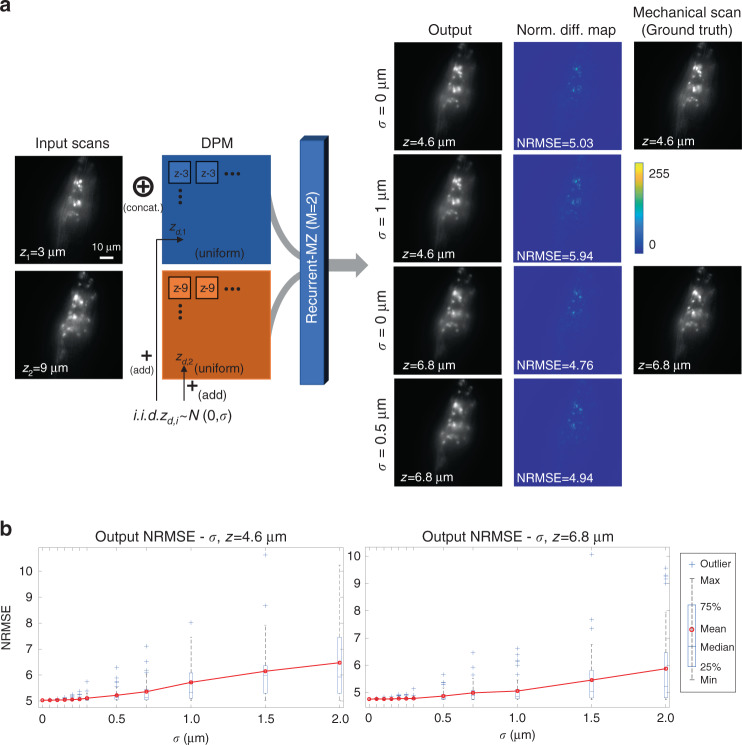
Stability test of Recurrent-MZ inference. **a** An additive Gaussian noise with zero mean and a standard variance of *σ* was injected into each DPM to test the stability of Recurrent-MZ inference. The output images and difference maps (with respect to ground truth) with no injected noise (*σ* = 0) and with different levels of noise injection are shown. **b** The NRMSE-*σ* boxplots for Recurrent-MZ output images at *z* = 4.6 μm and *z* = 6.8 μm are reported. NRMSE values were calculated over 50 random tests. The difference maps were normalized by the maximum difference between the input images and the ground truth

The results of this noise analysis reveal that, as illustrated in Fig. 5b, the output images of Recurrent-MZ (*M* = 2) at *z* = 4.6 μm degrade as the variance of the injected noise increases, as expected. However, even at a relatively significant noise level, where the microscope stage or sample drift is represented with a standard variation of *σ* = 1 μm (i.e., 2.5-fold of the objective lens depth-of-field, 0.4 μm), Recurrent-MZ inference successfully matches the ground truth with an NRMSE of 5.94; for comparison, the baseline inference (with *σ* = 0 μm) has an NRMSE of 5.03. The same conclusion also holds for output images at *z* = 6.8 μm, which highlights the resilience of Recurrent-MZ framework against axial scanning errors and/or uncontrolled drifts in the sample/stage.

### Permutation invariance of Recurrent-MZ

**Fig. 6:**
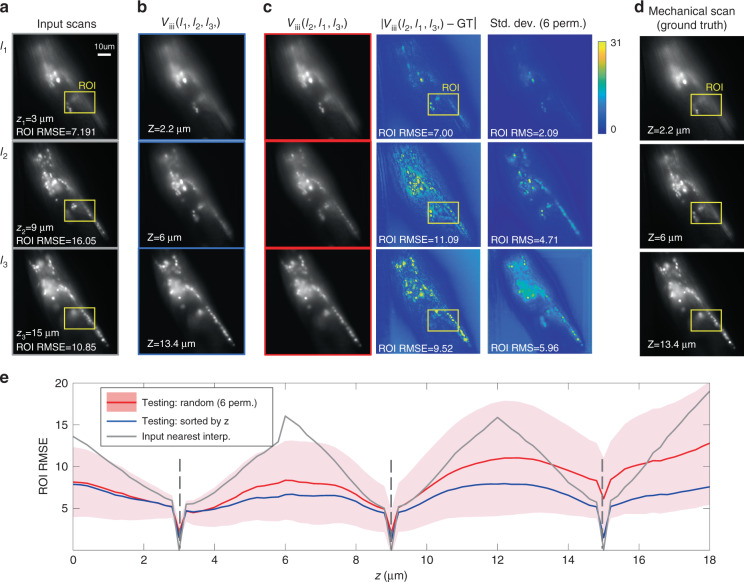
Permutation invariance of Recurrent-MZ to the input images. Recurrent-MZ was trained with inputs (*M* = 3) sorted by *z* and tested on new samples with both inputs sorted by *z* as well as 6 random permutations of the same inputs to test its permutation invariance. **a** The input images sorted by *z*, and the RMSE values between the ground truth image and the corresponding nearest input image are shown. **b** The Recurrent-MZ outputs of the input sequence (*I*_1_, *I*_2_, *I*_3_), **c** the test outputs with input sequence (*I*_2_, *I*_1_, *I*_3_), the corresponding difference maps and the pixel-wise standard deviation over all the 6 random permutations, **d** the ground truth images obtained by mechanical scanning through the same sample, acquired with an axial spacing of 0.2 μm, **e** red solid line: the average RMSE of the outputs of randomly permuted input images; pink shadow: the standard deviation RMSE of the outputs of randomly permuted input images; blue solid line: the RMSE of the output of input images sorted by *z*; gray solid line: the RMSE value of the nearest interpolation using the input images, calculated with respect to the ground truth images. Gray dashed lines (vertical) indicate the axial positions of input images. RMSE and RMS values were calculated based on the yellow highlighted ROIs. The range of grayscale images is 255, while that of the standard variance images is 31

Next, we focused on *post hoc* interpretation^43,44^ of the Recurrent-MZ framework, *without* any modifications to its design or the training process. For this, we explored to see if Recurrent-MZ framework exhibits *permutation invariance*, i.e.,

$$V_M\left( {I_1,I_2, \cdots ,I_M} \right) = V_M\left( {I_{i_1},I_{i_2}, \cdots ,I_{i_M}} \right),\;\forall \left( {i_1,i_2, \cdots ,i_M} \right) \in S_M$$

where *S*_*M*_ is the permutation group of *M*. To explore the permutation invariance of Recurrent-MZ (see Fig. 6), the test set’s input images were randomly permuted, and fed into the Recurrent-MZ (*M* = 3), which was *solely trained with input images sorted by z*. We then quantified Recurrent-MZ outputs over all the 6 permutations of the *M* = 3 input images, using the average RMSE (*μ*_*RMSE*_) and the standard deviation of the RMSE (*σ*_*RMSE*_), calculated with respect to the ground truth image *I*:

$$\mu _{RMSE} = \frac{1}{6}\mathop {\sum}\limits_{\left( {i_1,i_2,i_3} \right) \in S_3} {{\mathrm{RMSE}}} \left( {{\mathrm{V}}_{{\mathrm{iii}}}\left( {I_{i_1},I_{i_2},I_{i_3}} \right),\,I} \right)$$

$$\sigma _{RMSE} = \sqrt {\frac{1}{6}\mathop {\sum}\limits_{\left( {i_1,i_2,i_3} \right) \in S_3} {\left( {{\mathrm{RMSE}}\left( {{\mathrm{V}}_{{\mathrm{iii}}}\left( {I_{i_1},I_{i_2},I_{i_3}} \right),\,I} \right) - \mu _{RMSE}} \right)^2} }$$

where *RMSE*(*I*, *J*) gives the RMSE between image *I* and *J*. In Fig. 6e, the red line indicates the average RMSE over 6 permutations and the pink shaded region indicates the standard deviation of RMSE over these 6 permutations. RMSE and RMS values were calculated based on the yellow highlighted regions of interest (ROIs) in Fig. 6. Compared with the blue line in Fig. 6e, which corresponds to the output of the Recurrent-MZ with the inputs sorted by *z*, the input image permutation results highlight the success of Recurrent-MZ with different input image sequences, despite being trained solely by depth sorted inputs. In contrast, *non-recurrent* CNN architectures, such as 3D U-Net^45^, inevitably lead to input permutation instability as they require a fixed length and sorted input sequences; this failure of *non-recurrent* CNN architectures is illustrated in Fig. S5.

We also explored different training schemes to further improve the permutation invariance of Recurrent-MZ, including training with input images sorted in descending order by the relative distance (*dz*) to the output plane as well as randomly sorted input images. As shown in Fig. S6, the Recurrent-MZ trained with input images that are sorted by depth, *z*, achieves the best inference performance, indicated by an NRMSE of 4.03, whereas incorporating randomly ordered inputs in the training phase results in the best generalization for different input image permutations. The analyses reported in Fig. S6 further highlight the impact of different training schemes on the inference quality and the permutation invariance feature of the resulting trained Recurrent-MZ network.

### Repetition invariance of Recurrent-MZ

**Fig. 7:**
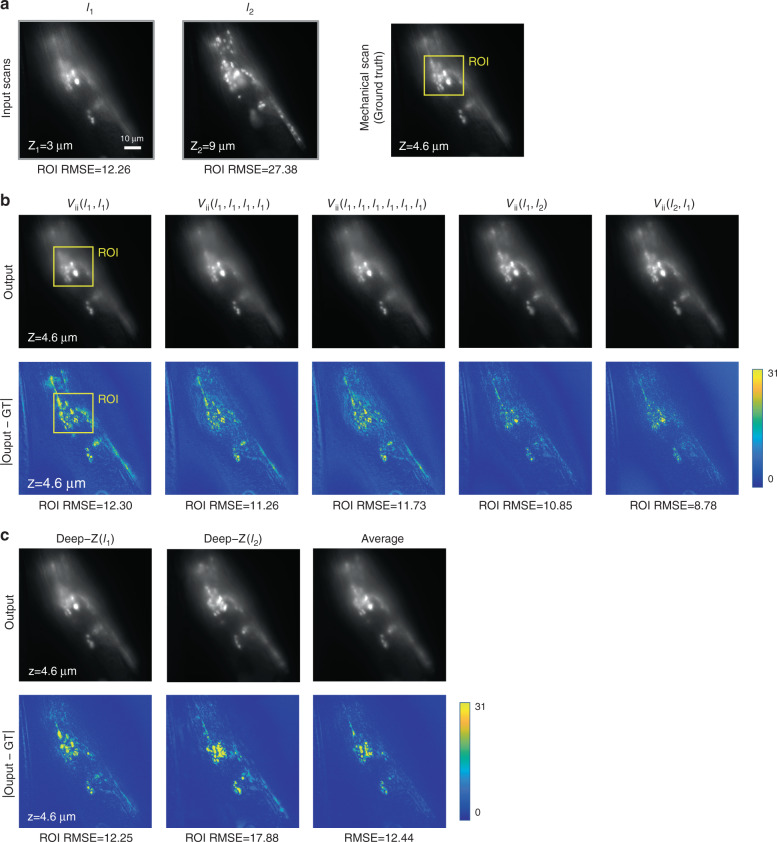
Repetition invariance of Recurrent-MZ. Recurrent-MZ was trained with inputs (*M* = 2) sorted by their relative distances (*dz*) to the output plane, but tested on a new sample by repeatedly feeding the input image (*I*_1_) to test its repetition invariance. **a** The input images and the ground truth image obtained by mechanical scanning (with an axial spacing of 0.2 μm), **b** the Recurrent-MZ outputs and the corresponding difference maps of repeated *I*_1_, i.e., *V*_ii_(*I*_1_, *I*_1_), *V*_ii_(*I*_1_, *I*_1_, *I*_1_, *I*_1_) and *V*_ii_(*I*_1_, *I*_1_, *I*_1_, *I*_1_, *I*_1_, *I*_1_) as well as *V*_ii_(*I*_1_, *I*_2_) and *V*_ii_(*I*_2_, *I*_1_), **c** the outputs and corresponding difference maps of Deep-Z with a single input image (*I*_1_ or *I*_2_), and the pixel-wise average of Deep-Z(*I*_1_) and Deep-Z(*I*_2_). All RMSE values are calculated based on the region of interest (ROI) marked by the yellow box. The range of grayscale images is 255 while that of the standard variance images is 31

Next, we explored to see if Recurrent-MZ framework exhibits repetition invariance. Figure 7 demonstrates the repetition invariance of Recurrent-MZ when it was repeatedly fed with input image *I*_1_. The output images of Recurrent-MZ in Fig. 7b show its consistency for 2, 4 and 6 repetitions of *I*_1_, i.e., *V*_ii_(*I*_1_, *I*_1_), *V*_ii_ (*I*_1_, *I*_1_, *I*_1_, *I*_1_) and *V*_ii_(*I*_1_, *I*_1_, *I*_1_, *I*_1_, *I*_1_, *I*_1_), which resulted in an RMSE of 12.30, 11.26, and 11.73, respectively. Although Recurrent-MZ was never trained with repeated input images, its recurrent scheme still demonstrates the correct propagation under repeated inputs of the same 2D plane. When compared with the output of Deep-Z (i.e., Deep*-Z*(*I*_1_)) shown in Fig. 7c, Recurrent-MZ, with a single input image or its repetitions, exhibits comparable reconstruction quality. Figure S7 also presents a similar comparison when *M* = 3, further supporting the same conclusion.

While for a single input image (*I*_1_ or its repeats) the blind inference performance of Recurrent-MZ is on par with Deep-Z(*I*_1_), the incorporation of multiple input planes gives a superior performance to Recurrent-MZ over Deep-Z. As shown in the last two columns of Fig. 7b, by adding another depth image, *I*_2_, the output of Recurrent-MZ is significantly improved, where the RMSE decreased to 8.78; this represents a better inference performance compared to Deep-Z(*I*_1_) and Deep-Z(*I*_2_) as well as the average of these two Deep-Z outputs (see Fig. 7b, c). The same conclusion is further supported in Fig. S7b, c for *M* = 3, demonstrating that Recurrent-MZ is able to outperform Deep-Z even if all of its M input images are individually processed by Deep-Z and averaged, showing the superiority of the presented recurrent inference framework.

### Demonstration of cross-modality volumetric imaging: wide-field to confocal

**Fig. 8:**
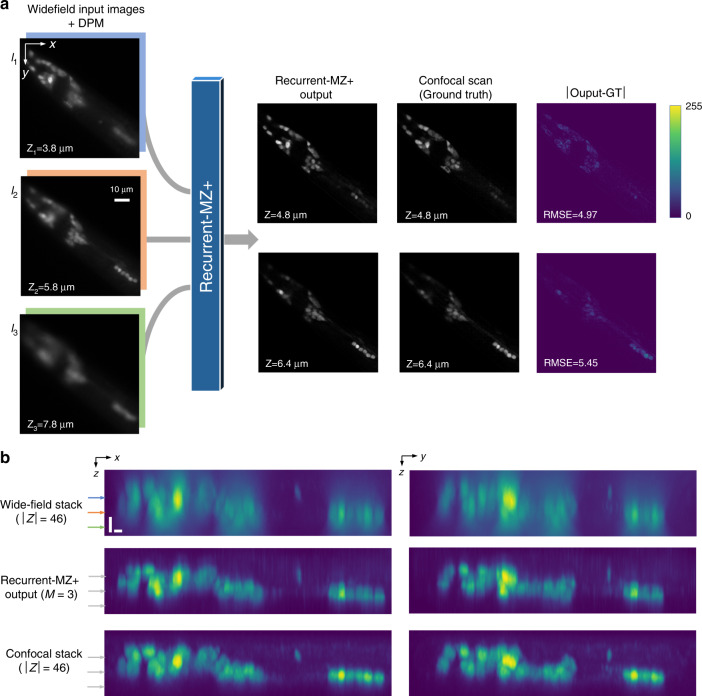
Wide-field to confocal: cross-modality volumetric imaging using Recurrent-MZ+. **a** Recurrent-MZ+ takes in *M* = 3 wide-field input images along with the corresponding DPMs, and rapidly outputs an image at the designated/desired axial plane, matching the corresponding confocal scan of the same sample plane. **b** Maximum intensity projection (MIP) side views (*x*–*z* and *y*–*z*) of the wide-field (46 image scans), Recurrent-MZ+ (*M* = 3) and the confocal ground truth image stack. Each scale bar is 2 μm. Horizontal arrows in (**b**) mark the axial planes of *I*_1_, *I*_2_ and *I*_3_. Also see Video S3

The presented Recurrent-MZ framework can also be applied to perform cross-modality volumetric imaging, e.g., from wide-field to confocal, where the network takes in a few wide-field 2D fluorescence images (input) to infer at its output a volumetric image stack, matching the fluorescence images of the same sample obtained by a confocal microscope; we termed this cross-modality image transformation framework as Recurrent-MZ+. To experimentally demonstrate this unique capability, Recurrent-MZ+ was trained using wide-field (input) and confocal (ground truth) image pairs corresponding to *C. elegans* samples (see the Materials and Methods section for details). Figure 8 and Movie S3 report blind-testing results on new images never used in the training phase. In Fig. 8, *M* = 3 wide-field images captured at *z* = 2.8, 4.8, and 6.8 μm were fed into Recurrent-MZ+ as input images and were virtually propagated onto axial planes from 0 to 9 μm with 0.2 μm spacing; the resulting Recurrent-MZ+ output images provided a very good match to the corresponding confocal 3D image stack obtained by mechanical scanning (also see Movie S3). Figure 8b further illustrates the maximum intensity projection (MIP) side views (*x-z* and *y-z*), showing the high fidelity of the reconstructed image stack by Recurrent-MZ+ with respect to the mechanical confocal scans. In contrast to the wide-field image stack of the same sample (with 46 image scans), where only a few neurons can be recognized in the MIP views with deformed shapes, the reconstructed image stack by Recurrent-MZ+ shows substantially sharper MIP views using only *M* = 3 input images, and also mitigates the neuron deformation caused by the elongated wide-field PSF, providing a comparable image quality with respect to the confocal microscopy image stack (Fig. 8b).

## Discussion

We demonstrated a new deep learning-based volumetric imaging framework termed Recurrent-MZ enabled by a convolutional recurrent neural network, which significantly extends the DOF of the microscopy system from sparse 2D scanning, providing a 30-fold reduction in the number of required mechanical scans. Another advantage of Recurrent-MZ is that it does not require special optical components in the microscopy set-up or an optimized scanning strategy. Despite being trained with equidistant input scans, Recurrent-MZ successfully generalized to use input images acquired with a non-uniform axial spacing as well as unknown axial positioning errors, all of which demonstrate its robustness.

In a practical application, the users of Recurrent-MZ should select an optimum *M* to provide a balance between the inference image quality of the reconstructed sample volume and the imaging throughput. For example, it is possible to set a stopping threshold, *ϵ*, for the volumetric reconstruction improvement that is provided by adding another image/scan to Recurrent-MZ, in terms of the Euclidean distance from the volume which was reconstructed from previous images; stated differently, the scanning can stop when e.g., ‖*V*_*M*_(*I*_1_, ..,*I*_*M*_) − *V*_*M*−1_(*I*_1_,..., *I*_*M*−1_)‖_*F*_ ≤ *ϵ*, where ‖·‖_*F*_ defines the Frobenius norm.

Importantly, this study shows the first application of convolutional recurrent neural networks in microscopic image reconstruction, and also reveals the potential of RNNs in microscopic imaging when sequential image data are acquired. With regards to solving inverse problems in microscopic imaging, most existing deep learning-based methods are optimized for a single shot/image, whereas sequential shots are generally convenient to obtain and substantial sample information hides in their 3D distribution. Through the incorporation of sequential 2D scans, the presented Recurrent-MZ integrates the information of different input images from different depths to gain considerable improvement in the volumetric image quality and the output DOF. Furthermore, the success of cross-modality image transformations using Recurrent-MZ+ reveals its potential for a wide spectrum of biomedical and biological applications, where confocal microscopy is frequently utilized. Using just a few wide-field 2D input images corresponding to a volumetric sample, Recurrent-MZ+ is able to rapidly provide a 3D image stack that is comparable to confocal microscopic imaging of the same sample volume (see Fig. 8 and Movie S3), potentially avoiding time-consuming scanning and substantially increasing the 3D imaging throughput.

In contrast to 3D CNNs that generally require a fixed sampling grid (see e.g., the failure of 3D U-Net with non-uniform axial sampling in Fig. 4), the presented recurrent scheme is compatible with (1) input sequences of variable lengths, as shown in Fig. 7, and (2) input images at variable, non-uniform axial sampling, as shown in Figs. 4 and S4. In various imaging applications, where the 3D distribution of the fluorescent samples has a large axial variation, exhibiting significant spatial non-uniformity, Recurrent-MZ’s compatibility with variable sampling grids provides us significant flexibility and performance advantage over conventional 3D CNNs that demand a fixed sampling grid. Another interesting property that we demonstrated is the robustness of Recurrent-MZ inference to input image permutations (Fig. 6), which could lead to catastrophic failure modes for standard convolutional networks, as also illustrated in Fig. S5. For 3D microscopy modalities under random, interlaced or other specific scanning modes, where the captured images can hardly be sorted, this unique permutation invariance could empower the Recurrent-MZ framework to correctly utilize the information of the input image sequence regardless of its order. One potential future application that might benefit from the above discussed unique advantages of the Recurrent-MZ framework is 3D microscopic imaging with an isotropic PSF. In general, 3D imaging with isotropic resolution can be achieved through e.g., image fusion and deconvolution from multiple views^46–49^, during which several image stacks from different viewing angles are acquired to improve 3D image resolution. The presented Recurrent-MZ framework and the underlying core principles could potentially be applied to incorporate 2D images that are sparsely captured at a variable sampling grid under the spherical coordinate system, i.e., sampling the 3D object at variable depths from variable viewing angles. Such a learning approach that is based on Recurrent-MZ framework might significantly reduce the number of axial scans and viewing angles needed to achieve an isotropic 3D resolution.

In summary, Recurrent-MZ provides a rapid and flexible volumetric imaging framework with reduced number of axial scans, and opens up new opportunities in machine learning-based 3D microscopic imaging. The presented recurrent neural network structure could also be widely applicable to process sequential data resulting from various other 3D imaging modalities such as OCT, Fourier ptychographic microscopy, holography, structured illumination microscopy, among others.

## Materials and methods

### Sample preparation, image acquisition and dataset preparation

The *C. elegans* samples were firstly cultured and stained with GFP using the strain AML18. AML18 carries the genotype wtfIs3 [rab-3p::NLS::GFP+rab-3p::NLS::tagRFP] and expresses GFP and tagRFP in the nuclei of all the neurons. *C. elegans* samples were cultured on nematode growth medium seeded with OP50 E. Coli bacteria using standard conditions. During the imaging process, the samples were washed off the plates with M9 solution and anesthetized with 3 mM levamisole, and then mounted on slides seeded with 3% agarose.

The wide-field and confocal microscopy images of *C. elegans* were captured by an inverted scanning microscope (TCS SP8, Leica Microsystems), using a 63×/1.4NA objective lens (HC PL APO 63×/1.4NA oil CS2, Leica Microsystems) and a FITC filter set (excitation/emission wavelengths: 495 nm/519 nm), resulting in a DOF about 0.4 μm. A monochrome scientific CMOS camera (Leica DFC9000GTC-VSC08298) was used for wide-field imaging where each image has 1024 × 1024 pixels and 12-bit dynamic range; a photo-multiplier tube (PMT) recorded the confocal image stacks. For each FOV, 91 images with 0.2 μm axial spacing were recorded, where the starting position of the axial scan (*z* = 0 μm) was set on the boundary of each worm. A total of 100 FOVs were captured and exclusively divided into training, validation and testing datasets at the ratio of 41:8:1, respectively, where the testing dataset was strictly captured on distinct worms that were not used in training dataset.

The nanobead image dataset consists of wide-field microscopic images that were captured using 50 nm fluorescence beads with a Texas Red filter set (excitation/emission wavelengths: 589 nm/615 nm). The wide-field microscopy system consists of an inverted scanning microscope (TCS SP8, Leica Microsystems) and a 63×/1.4NA objective lens (HC PL APO 63×/1.4NA oil CS2, Leica Microsystems). The nanobeads were purchased from MagSphere (PSF-050NM RED), and ultrasonicated before dilution into the heated agar solution. ~1 mL diluted bead-agar solution was further mixed to break down the bead clusters and then a 2.5 µL droplet was pipetted onto a cover slip, spread and dried for imaging. Axial scanning was implemented and the system started to record images (*z* = 0 μm) when a sufficient number of nanobeads could be seen in the FOV. Each volume contains 101 images with 0.1 μm axial spacing. A subset of 400, 86 and 16 volumes were exclusively divided as training, validation and testing datasets.

Each captured image volume was first axially aligned using the ImageJ plugin ‘StackReg’^50^ for correcting the lateral stage shift and stage rotation. Secondly, an image with extended depth of field (EDF) was generated for each volume, using the ImageJ plugin ‘Extended Depth of Field’^51^. The EDF image was later used as a reference for the following image processing steps: (1) apply triangle thresholding to the EDF image to separate the background and foreground contents^38^, (2) draw the mean intensity from the background pixels as the shift factor, and the 99% percentile of the foreground pixels as the scale factor, (3) normalize the volume by the shift and scale factors. For Recurrent-MZ+, confocal image stacks were registered to their wide-field counterparts using the same feature-based registration method reported earlier^38^. Thirdly, training FOVs were cropped into small regions of 256 × 256 pixels without any overlap. Eventually, the data loader randomly selects *M* images from the volume with an axial spacing of Δ*z* = 6 μm (*C. elegans*) and Δ*z* = 3 μm (nanobeads) in both the training and testing phases.

### Network structure

Recurrent-MZ is based on a convolutional recurrent network^52^ design, which combines the advantages of both convolutional neural networks^39^ and recurrent neural networks in processing sequential inputs^53,54^. A common design of the network is formed by an encoder-decoder structure^55,56^, with the convolutional recurrent units applying to the latent domain^40,57–59^. Furthermore, inspired by the success of exploiting multiscale features in image translation tasks^60–62^, a sequence of cascaded encoder-decoder pairs is utilized to exploit and incorporate image features at different scales from different axial positions.

As shown in Fig. 1b, the output of last encoder block *x*_*k*−1_ is pooled and then fed into the *k*-th block, which can be expressed as

1 $$x_k = {\mathrm{ReLU}}\left( {{\mathrm{BN}}\left( {{\mathrm{Conv}}_{k,2}\left( {{\mathrm{ReLU}}\left( {{\mathrm{BN}}\left( {{\mathrm{Conv}}_{k,1}\left( {{\mathrm{P}}\left( {x_{k - 1}} \right)} \right)} \right)} \right)} \right)} \right)} \right)$$

where *P*(·) is the 2 × 2 max-pooling operation, BN(·) is batch normalization, ReLU(·) is the rectified linear unit activation function and Conv_*k,i*_(·) stands for the *i*-th convolution layer in the *k*-th encoder block. The convolution layers in all convolution blocks have a kernel size of 3 × 3, with a stride of 1, and the number of channels for Conv_*k*,1_ and Conv_*k*,2_ are 20 · 2^*k*−2^ and 20 · 2^*k*−1^, respectively. Then, *x*_*k*_ is sent to the recurrent block, where features from the sequential input images are recurrently integrated:

2 $$s_k = x_k + {\mathrm{Conv}}_{k,3}\left( {{\mathrm{RConv}}_{k}\left( {x_k} \right)} \right)$$

where RConv_*k*_(·) is the convolutional recurrent layer with kernels of 3 × 3 and a stride of 1, the Conv_*k,3*_(·) is a 1 × 1 convolution layer. Finally, at the decoder part, *s*_*k*_ is concatenated with the up-sampled output from last decoder convolution block, and fed into the k-th decoder block, so the output of k-th decoder block can be expressed as

3 $$y_k = {\mathrm{ReLU}}\left( {{\mathrm{BN}}\left( {{\mathrm{Conv}}_{k,5}\left( {{\mathrm{ReLU}}\left( {{\mathrm{BN}}\left( {{\mathrm{Conv}}_{k,4}\left( {{\mathrm{I}}\left( {y_{k - 1}} \right) \oplus s_k} \right)} \right)} \right)} \right)} \right)} \right)$$

where ⊕ is the concatenation operation, I(·) is the 2 × 2 up-sampling operation using nearest interpolation and Conv_*k,i*_(·) are the convolution layers of the *k*-th decoder block.

In this work, the gated recurrent unit (GRU)^63^ is used as the recurrent unit, i.e., the RConv(·) layer in Eq. (2) updates *h*_*t*_, given the input *x*_*t*_, through the following three steps:

4 $$f_t = \sigma \left( {W_f \ast x_t + U_f \ast h_{t - 1} + b_f} \right)$$

5 $$\widehat {h_t} = \tan \!{\mathrm{h}}\left( {W_h \ast x_t + U_h \ast \left( {f_t \odot h_{t - 1}} \right) + b_h} \right)$$

6 $$h_t = \left( {1 - f_t} \right) \odot h_{t - 1} + f_t \odot \widehat {h_t}$$

where *f*_*t*_, *h*_*t*_ are forget and output vectors at time step *t*, respectively, *W*_*f*_, *W*_*h*_, *U*_*f*_, *U*_*h*_ are the corresponding convolution kernels, *b*_*f*_, *b*_*h*_ are the corresponding biases, *σ* is the sigmoid activation function, * is the 2D convolution operation, and ⊙ is the element-wise multiplication. Compared with long short term memory (LSTM) network^64^, GRU entails fewer parameters but is able to achieve similar performance.

The discriminator (*D*) is a CNN consisting of five convolutional blocks and two dense layers. The *k-*th convolutional block has two convolutional layers with 20 · 2^*k*^ channels. A global average pooling layer compacts each channel before the dense layers. The first dense layer has 20 hidden units with ReLU activation function and the second dense layer uses a sigmoid activation function. The GAN structure and other details of both the generator and discriminator networks are reported in Fig. S8.

### Recurrent-MZ implementation

The Recurrent-MZ was written and implemented using TensorFlow 2.0. In both training and testing phases, a DPM is automatically concatenated with the input image by the data loader, indicating the relative axial position of the input plane to the desired output plane, i.e., the input in the training phase has dimensions of *M* × 256 × 256 × 2. Through varying the DPMs, Recurrent-MZ learns to digitally propagate inputs to any designated plane, and thus forming an output volume with dimensions of |*Z*| × 256 × 256.

The training loss of Recurrent-MZ is composed of three parts: (i) pixel-wise BerHu loss^65,66^, (ii) multiscale structural similarity index (MSSSIM)^67^, and (iii) the adversarial loss using the generative adversarial network (GAN)^68^ structure. Based on these, the total loss of Recurrent-MZ, i.e., *L*_*V*_, is expressed as

7 $$L_V = \alpha {\mathrm{BerHu}}\left( {\hat y,y} \right) + \beta {\mathrm{MSSSIM}}\left( {\hat y,y} \right) + \gamma \left[ {D\left( {\hat y} \right) - 1} \right]^2$$

$\hat y$ is the output image of the Recurrent-MZ, and *y* is the ground truth image for a given axial plane. *α*, *β*, *γ* are the hyperparameters, which were set as 3, 1 and 0.5, respectively. And the MSSSIM and BerHu losses are expressed as:

8 $${\mathrm{MSSSIM}}\left( {x,\,y} \right) = \left[ {\frac{{2\mu _{x_M}\mu _{y_M} + C_1}}{{\mu _{x_M}^2 + \mu _{y_M}^2 + C_1}}} \right]^{\alpha _M} \times \mathop {\prod}\limits_{j = 1}^M {\left[ {\frac{{2\sigma _{x_j}\sigma _{y_j} + C_2}}{{\sigma _{x_j}^2 + \sigma _{y_j}^2 + C_2}}} \right]^{\beta _j}\left[ {\frac{{\sigma _{x_jy_j}^2 + C_3}}{{\sigma _{x_j}\sigma _{y_j} + C_3}}} \right]^{\gamma _j}}$$

9 $${\mathrm{BerHu}}\left( {x,y} \right) = \mathop {\sum}\limits_{\mathop {{m,n}}\limits_{\left| {x\left( {m,n} \right) - y\left( {m,n} \right)} \right| \le c} } {\left| {x\left( {m,n} \right) - y\left( {m,n} \right)} \right|} + \mathop {\sum}\limits_{\mathop {{m,n}}\limits_{\left| {x\left( {m,n} \right) - y\left( {m,n} \right)} \right| > c} } {\frac{{\left[ {x\left( {m,n} \right) - y\left( {m,n} \right)} \right]^2 + c^2}}{{2c}}}$$

*x*_*j*_, *y*_*j*_ are 2^*j*−1^ down-sampled images of *x*,*y*, respectively, $\mu _x,\,\sigma _x^2$ denote the mean and variance of *x*, respectively, and $\sigma _{xy}^2$ denotes the covariance between *x* and *y*. *x*(*m*,*n*) is the intensity value at pixel (*m*,*n*) of image *x*. *α*_*M*_, *β*_*j*_, *γ*_*j*_, *C*_*i*_ are empirical constants^67^ and *c* is a constant set as 0.1. BerHu and MSSSIM losses provide a structural loss term, in addition to the adversarial loss, focusing on the high-level image features. The combination of SSIM or MSSSIM evaluating regional or global similarity, and a pixel-wise loss term with respect to the ground truth (such as L1, L2, Huber and BerHu) has been shown to improve network performance in image translation and restoration tasks^69^.

The loss for the discriminator *L*_*D*_ is defined as:

10 $$L_D = \frac{1}{2}D\left( {\hat y} \right)^2 + \frac{1}{2}\left[ {D\left( y \right) - 1} \right]^2$$

where *D* is the discriminator of the GAN framework. An Adam optimizer^70^ with an initial learning rate 10^−5^ was employed for stochastic optimization.

The training time on a PC with Intel Xeon W-2195 CPU, 256 GB RAM and one single NVIDIA RTX 2080 Ti graphic card is about 3 days. After optimization for mixed precision and parallel computation, the image reconstruction using Recurrent-MZ (*M* = 3) takes ~0.15 s for an output image of 1024 × 1024, and ~3.42s for a volume of 101 × 1024 × 1024 pixels.

### The implementation of Deep-Z

The Deep-Z network, used for comparison purposes, is identical as in ref. ^38^, and was trained and tested on the same dataset as Recurrent-MZ using the same machine. The loss function, optimizer and hyperparameter settings were also identical to ref. ^38^. Due to the single-scan propagation of Deep-Z, the training range is $\frac{1}{M}$ of that of Recurrent-MZ, depending on the value of *M* used in the comparison. The reconstructed volumes over a large axial range, as presented in the manuscript, were axially stacked using *M* non-overlapping volumes, which were propagated from different input scans and covered $\frac{1}{M}$ of the total axial range. The Deep-Z reconstruction time for a 1024 × 1024 output image on the same machine as Recurrent-MZ is ~0.12 s.

### The implementation of 3D U-Net

For each input sequence of *M* × 256 × 256 × 2 (the second channel is the DPM), it was reshaped as a tensor of 256 × 256 × (2*M*) and fed into the 3D U-Net^45^. When permuting the *M* input scans, the DPMs always follow the corresponding images/scans. The number of channels at the last convolutional layer of each down-sampling block is 60 · 2^*k*^ and the convolutional kernel is 3 × 3 × 3. The network structure is the same as reported in ref. ^45^. The other training settings, such as the loss function and optimizer are similar to Recurrent-MZ. The reconstruction time (*M* = 3) for an output image of 1024 × 1024 on the same machine (Intel Xeon W-2195 CPU, 256 GB RAM and one single NVIDIA RTX 2080 Ti graphic card) is ~0.2 s.

## Supplementary information

Supplementary Video S1

Supplementary Video S2

Supplementary Video S3

Supplementary Information

## References

[1] Pawley, J. B. *Handbook of Biological Confocal Microscopy*. 3rd edn. (Springer-Verlag, New York, 2006).

[2] Denk W, Strickler JH, Webb WW (1990). Two-photon laser scanning fluorescence microscopy. Science.

[3] Horton NG (2013). In vivo three-photon microscopy of subcortical structures within an intact mouse brain. Nat. Photonics.

[4] Huang D (1991). Optical coherence tomography. Science.

[5] Haeusler G, Lindner MW (1998). “Coherence radar” and “spectral radar”—new tools for dermatological diagnosis. J. Biomed. Opt..

[6] Fercher AF (1995). Measurement of intraocular distances by backscattering spectral interferometry. Opt. Commun..

[7] Santi PA (2011). Light sheet fluorescence microscopy: a review. J. Histochem. Cytochem..

[8] Prabhat P (2004). Simultaneous imaging of different focal planes in fluorescence microscopy for the study of cellular dynamics in three dimensions. IEEE Trans. NanoBiosci..

[9] Johnson C (2019). Continuous focal translation enhances rate of point-scan volumetric microscopy. Opt. Express.

[10] Abrahamsson S (2013). Fast multicolor 3D imaging using aberration-corrected multifocus microscopy. Nat. Methods.

[11] Bouchard MB (2015). Swept confocally-aligned planar excitation (SCAPE) microscopy for high-speed volumetric imaging of behaving organisms. Nat. Photonics.

[12] Nakano A (2002). Spinning-disk confocal microscopy—a cutting-edge tool for imaging of membrane traffic. Cell Struct. Funct..

[13] Badon A (2019). Video-rate large-scale imaging with Multi-Z confocal microscopy. Optica.

[14] Li HY (2019). Fast, volumetric live-cell imaging using high-resolution light-field microscopy. Biomed. Opt. Express.

[15] Martínez-Corral M, Javidi B (2018). Fundamentals of 3D imaging and displays: a tutorial on integral imaging, light-field, and plenoptic systems. Adv. Opt. Photonics.

[16] Song A (2017). Volumetric two-photon imaging of neurons using stereoscopy (vTwINS). Nat. Methods.

[17] Chen XL (2017). Volumetric chemical imaging by stimulated Raman projection microscopy and tomography. Nat. Commun..

[18] Lu RW (2017). Video-rate volumetric functional imaging of the brain at synaptic resolution. Nat. Neurosci..

[19] Pascucci M (2019). Compressive three-dimensional super-resolution microscopy with speckle-saturated fluorescence excitation. Nat. Commun..

[20] Fang LY (2013). Fast acquisition and reconstruction of optical coherence tomography images via sparse representation. IEEE Trans. Med. Imaging.

[21] Wen CY (2019). Compressive sensing for fast 3-D and random-access two-photon microscopy. Opt. Lett..

[22] Beck A, Teboulle M (2009). A fast iterative shrinkage-thresholding algorithm for linear inverse problems. SIAM J. Imaging Sci..

[23] Boyd S (2011). Distributed optimization and statistical learning via the alternating direction method of multipliers. Found. Trends Mach. Learn..

[24] de Haan K (2020). Deep-learning-based image reconstruction and enhancement in optical microscopy. Proc. IEEE.

[25] Rivenson Y (2017). Deep learning microscopy. Optica.

[26] Wang HD (2019). Deep learning enables cross-modality super-resolution in fluorescence microscopy. Nat. Methods.

[27] Nehme E (2018). Deep-STORM: super-resolution single-molecule microscopy by deep learning. Optica.

[28] Rivenson Y (2019). Virtual histological staining of unlabelled tissue-autofluorescence images via deep learning. Nat. Biomed. Eng..

[29] Bayramoglu, N. et al. Towards virtual H&E staining of hyperspectral lung histology images using conditional generative adversarial networks. In: *Proc. 2017 IEEE International Conference on Computer Vision Workshops (ICCVW)* 64–71 (IEEE, Venice, Italy, 2017).

[30] Christiansen EM (2018). *In silico* labeling: predicting fluorescent labels in unlabeled images. Cell.

[31] Ounkomol C (2018). Label-free prediction of three-dimensional fluorescence images from transmitted-light microscopy. Nat. Methods.

[32] Rivenson Y (2019). PhaseStain: the digital staining of label-free quantitative phase microscopy images using deep learning. Light.: Sci. Appl..

[33] Wu YC (2018). Extended depth-of-field in holographic imaging using deep-learning-based autofocusing and phase recovery. Optica.

[34] Rivenson Y (2018). Phase recovery and holographic image reconstruction using deep learning in neural networks. Light.: Sci. Appl..

[35] Nguyen T (2018). Deep learning approach for Fourier ptychography microscopy. Opt. Express.

[36] Pinkard H (2019). Deep learning for single-shot autofocus microscopy. Optica.

[37] Luo YL (2021). Single-shot autofocusing of microscopy images using deep learning. ACS Photonics.

[38] Wu YC (2019). Three-dimensional virtual refocusing of fluorescence microscopy images using deep learning. Nat. Methods.

[39] Barbastathis G, Ozcan A, Situ G (2019). On the use of deep learning for computational imaging. Optica.

[40] Choy, C. B. et al. 3D-R2N2: a unified approach for single and multi-view 3D object reconstruction. In: *Proc. 14th European Conference on Computer Vision (ECCV) 2016*. 628-644. (Springer, Amsterdam, The Netherlands, 2016)

[41] Kar, A., Häne, C. & Malik, J. Learning a multi-view stereo machine. In: *Proc. 31st International Conference on Neural Information Processing Systems* (ACM, Long Beach, CA, USA, 2017).

[42] Petrov PN, Moerner WE (2020). Addressing systematic errors in axial distance measurements in single-emitter localization microscopy. Opt. Express.

[43] Montavon G, Samek W, Müller KR (2018). Methods for interpreting and understanding deep neural networks. Digital Signal Process..

[44] Selvaraju, R. R. et al. Grad-CAM: visual explanations from deep networks via gradient-based localization. In: *Proc. 2017 IEEE International Conference on Computer Vision (ICCV)*. (IEEE, Venice, Italy, 2017).

[45] Çiçek, Ö. et al. 3D U-Net: learning dense volumetric segmentation from sparse annotation. In: *Proc. 19th International Conference on Medical Image Computing and Computer-Assisted Intervention – MICCAI 2016*. 424–432. (Springer, Athens, Greece, 2016).

[46] Chhetri RK (2015). Whole-animal functional and developmental imaging with isotropic spatial resolution. Nat. Methods.

[47] Kumar A (2014). Dual-view plane illumination microscopy for rapid and spatially isotropic imaging. Nat. Protoc..

[48] Wu YC (2013). Spatially isotropic four-dimensional imaging with dual-view plane illumination microscopy. Nat. Biotechnol..

[49] Swoger J (2007). Multi-view image fusion improves resolution in three-dimensional microscopy. Opt. Express.

[50] Thevenaz P, Ruttimann UE, Unser M (1998). A pyramid approach to subpixel registration based on intensity. IEEE Trans. Image Process..

[51] Forster B (2004). Complex wavelets for extended depth-of-field: a new method for the fusion of multichannel microscopy images. Microsc. Res. Tech..

[52] Shi, X. J. et al. Convolutional LSTM network: a machine learning approach for precipitation nowcasting. In: *Proc. 28th International Conference on Neural Information Processing Systems*. (ACM, Montreal, Quebec, Canada, 2015).

[53] Graves A (2009). A novel connectionist system for unconstrained handwriting recognition. IEEE Trans. Pattern Anal. Mach. Intell..

[54] Gregor, K. et al. DRAW: a recurrent neural network for image generation. In *Proc. 32nd Internnational Conference on Machine Learning 2015*. 1462-1471. (PMLR, Lille, France, 2015).

[55] Sharma, A., Grau, O. & Fritz, M. VConv-DAE: deep volumetric shape learning without object labels. In *Proc. 14th European Conference on Computer Vision (ECCV) 2016*. 236-250. (Springer, Amsterdam, The Netherlands, 2016).

[56] Kingma, D. P. & Welling, M. Auto-encoding variational bayes. Preprint at http://arxiv.org/abs/1312.6114 (2014).

[57] Wang, W. Y. et al. Shape inpainting using 3D generative adversarial network and recurrent convolutional networks. In: *Proc. 2017 IEEE International Conference on Computer Vision (ICCV)*. 2317–2325. (IEEE, Venice, Italy, 2017).

[58] Chen, J. X. et al. Combining fully convolutional and recurrent neural networks for 3D biomedical image segmentation. In: *Proc. 30th International Conference on Neural Information Processing Systems*. (ACM, Barcelona, Spain, 2016).

[59] Tseng, K. L. et al. Joint sequence learning and cross-modality convolution for 3D biomedical segmentation. In: *Proc. 2017 IEEE Conference on Computer Vision and Pattern Recognition (CVPR)*. 3739–3746. (IEEE, Honolulu, HI, 2017).

[60] Ronneberger, O., Fischer, P. & Brox, T. U-Net: convolutional networks for biomedical image segmentation. Preprint at http://arxiv.org/abs/1505.04597 (2015).

[61] Zhou ZW (2020). UNet++: redesigning skip connections to exploit multiscale features in image segmentation. IEEE Trans. Med. Imaging.

[62] Liu, P. J. et al. Multi-level wavelet-CNN for image restoration. In: *Proc. 2018 IEEE/CVF Conference on Computer Vision and Pattern Recognition Workshops (CVPRW)*. 886–88609. (IEEE, Salt Lake City, UT, USA, 2018).

[63] Cho, K. et al. Learning phrase representations using RNN encoder-decoder for statistical machine translation. Preprint at http://arxiv.org/abs/1406.1078 (2014).

[64] Hochreiter S, Schmidhuber J (1997). Long short-term memory. Neural Comput..

[65] Owen, A. B. A robust hybrid of lasso and ridge regression. in *Prediction and Discovery* (eds Verducci, J. S., Shen, X. T. & Lafferty, J.) 59–71 (American Mathematical Society, Providence, Rhode Island, 2007).

[66] Laina, I. et al. Deeper depth prediction with fully convolutional residual networks. Preprint at http://arxiv.org/abs/1606.00373 (2016).

[67] Wang, Z., Simoncelli, E. P. & Bovik, A. C. Multiscale structural similarity for image quality assessment. In: *Proc. 37th Asilomar Conference on Signals, Systems & Computers*. (IEEE, Pacific Grove, CA, USA, 2003, 1398–1402).

[68] Goodfellow, I. J. et al. Generative adversarial nets. In: *Proc. 27th International Conference on Neural Information Processing Systems*. (ACM, Montreal, Quebec, Canada, 2014).

[69] Zhao H (2017). Loss functions for image restoration with neural networks. IEEE Trans. Computational Imaging.

[70] Kingma, D. P. & Ba, J. Adam: a method for stochastic optimization. Preprint at http://arxiv.org/abs/1412.6980 (2017).

