# Supplementary material for: Recurrent neural network-based volumetric fluorescence microscopy
Source: Light Sci Appl. 2021 Mar 23;10:62. doi: 10.1038/s41377-021-00506-9 (PMC7985192; doi:10.1038/s41377-021-00506-9)
Supplement: Supplementary file 4 — Supplementary Information [file 41377_2021_506_MOESM4_ESM.pdf]

Supplementary Information for:

Recurrent neural network-based volumetric fluorescence microscopy

Authors:

Luzhe Huang[1,2,3], Hanlong Chen[1], Yilin Luo[1], Yair Rivenson[1,2,3], Aydogan Ozcan[1,2,3,4]*

Affiliations:

[1] Electrical and Computer Engineering Department, University of California, Los Angeles, California 90095, USA

[2] Bioengineering Department, University of California, Los Angeles, California 90095, USA

[3] California Nano Systems Institute (CNSI), University of California, Los Angeles, California 90095, USA

[4] David Geffen School of Medicine, University of California, Los Angeles, California 90095, USA

Email addresses of all the authors:

lzhaung0324@ucla.edu, hanlongchen96@gmail.com, yilinluo@ucla.edu, rivensonyair@ucla.edu, ozcan@ucla.edu

Correspondence:

Dr. Aydogan Ozcan

Address: 420 Westwood Plaza, Engr. IV 68-119, UCLA, Los Angeles, CA 90095, USA

Tel: +1(310)825-0915

Fax: +1(310)206-4685

ozcan@ucla.edu

Supplementary Note 1: Comparison between Recurrent-MZ and interpolation algorithms

In general, the forward imaging model of wide-field microscopy can be expressed as[1]:

$$g_z = \mathcal{P}\{H_z H f\} + \mathcal{T}$$

where $\mathcal{P}, \mathcal{T}$ stand for a Poisson random process and noise terms caused by e.g., thermal noise and various sources of imperfections in the imaging system, respectively; $\mathcal{T}$ is often modelled as an additive Gaussian white noise. H is a circulant matrix representing 3D convolution with the system's PSF, H_z is a down-sampling matrix resulting in the sparse input image scans at axial planes $z_i, i = 1,2,\cdots, M$, and g_z, f are the vectorized output image and the object, respectively. Recurrent-MZ solves the volumetric image propagation problem to reconstruct images $g_{\tilde{z}}, \tilde{z} \in Z$ within a sample volume using an input sequence $g_{z_i}, i = 1,2,\cdots, M$. In contrast, standard interpolation algorithms simply model g_z as a specific continuous function, e.g., Hermite polynomial, linear function, etc. within each interval between given data nodes. Existing interpolation algorithms[2–4] are therefore incapable of *physically-correct* volumetric propagation of fluorescence images. As shown in Fig. S1, Recurrent-MZ matches the corresponding ground truth images very well, while various forms of interpolation methods fail and start to hallucinate features that are not real. Figure S1(b) further illustrates that Recurrent-MZ (red line) outperforms these interpolation algorithms in terms of both RMSE and PSNR metrics.

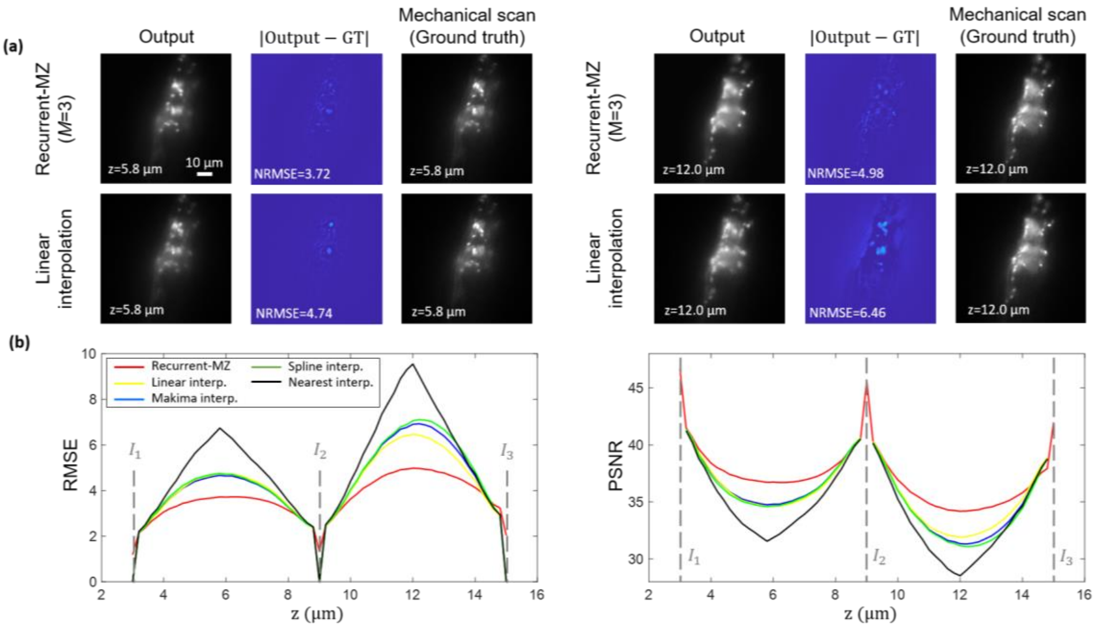

Fig. S1 Comparison of Recurrent-MZ and various interpolation methods. Four different 3D interpolation algorithms, including linear, modified Akima (makima)[3,4], spline (cubic) and nearest interpolation, were applied to the same input images used by Recurrent-MZ. Recurrent-MZ outperforms these interpolation algorithms in terms of both RMSE and PSNR metrics.

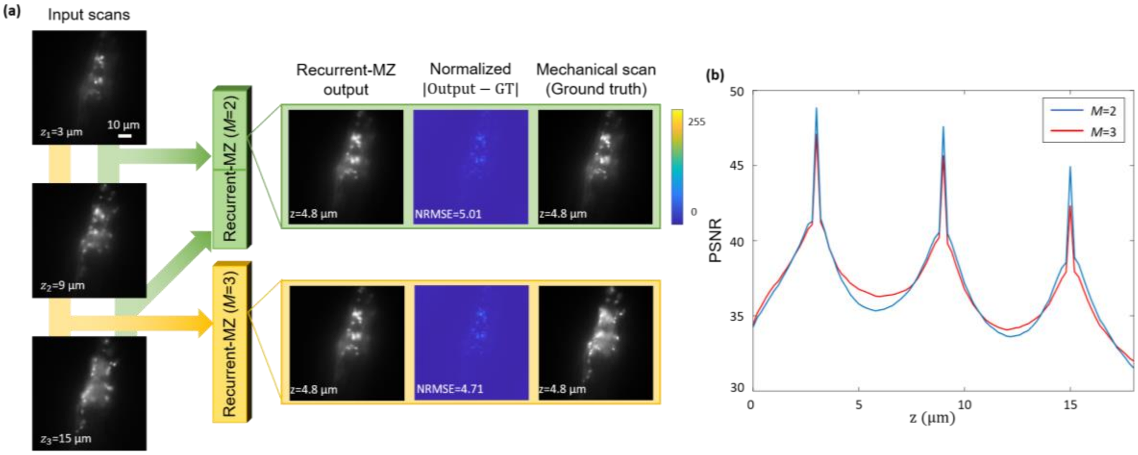

Fig. S2 Comparison of the use of different M in Recurrent-MZ. (a) The two networks were trained on the same dataset. For the testing sequence with 3

input images, the network with $M = 2$ takes in the 2 nearest input images to each output plane, while the network with $M = 3$ always takes in all 3 input images. (b) The PSNR values of the output images are calculated with respect to the corresponding ground truth image. Blue: Outputs of the Recurrent-MZ (*M*=2); Red: Outputs of the Recurrent-MZ (*M*=3).

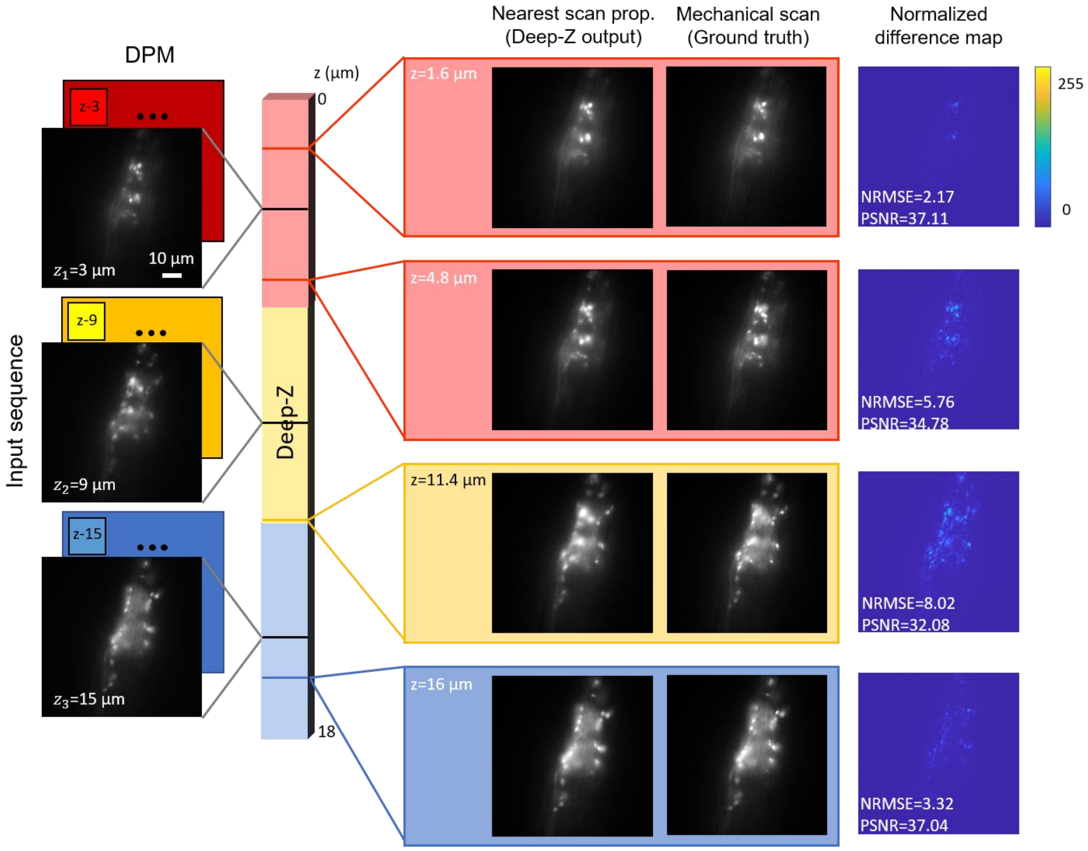

Fig. S3 Volumetric image reconstruction using Deep-Z on a *C. elegans* sample. Deep-Z takes in a single input image to infer an output image at the designated plane, as indicated by the color of each output box. See Fig. 2 for a comparison against Recurrent-MZ.

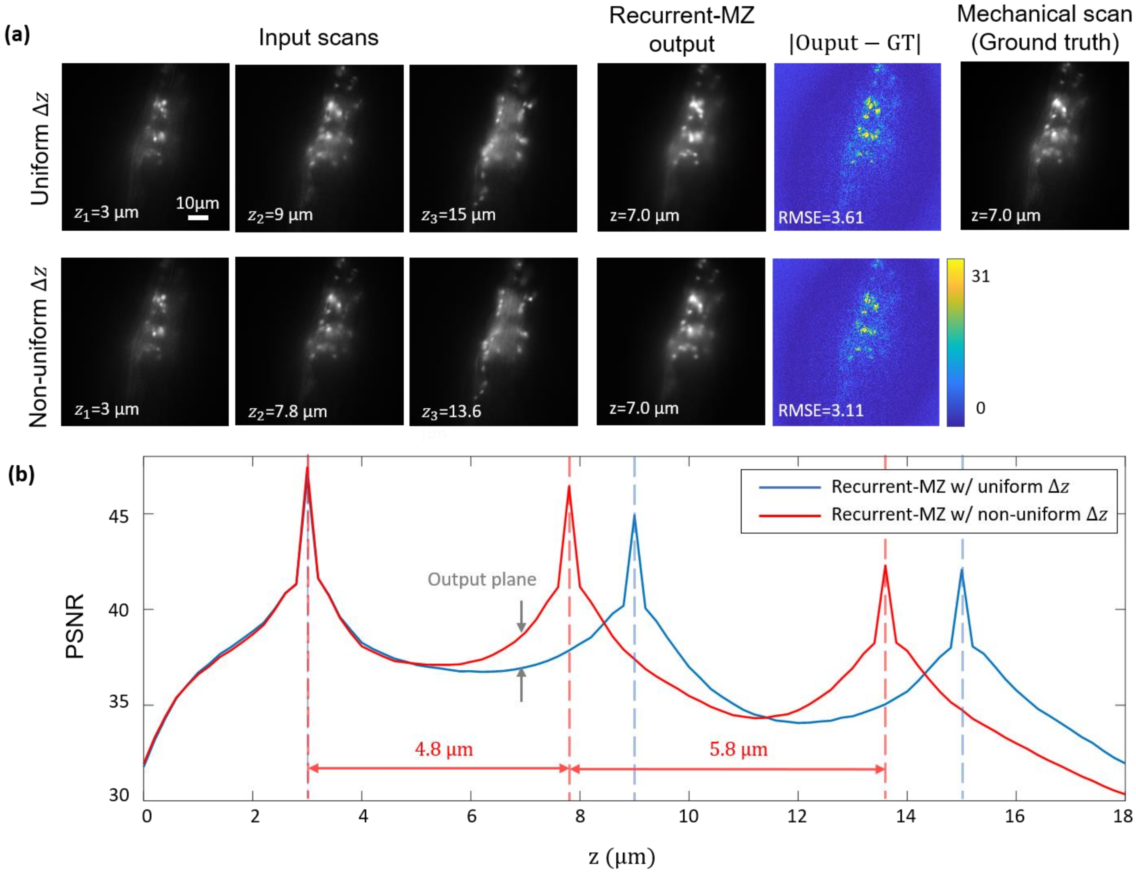

Fig. S4 Generalization of Recurrent-MZ to non-uniformly spaced input images. (a) Recurrent-MZ was trained on *C. elegans* samples with equidistant inputs (M=3, $\Delta z = 6$ µm), and blindly tested on both uniformly sampled and non-uniformly sampled input images of new samples. (b) The PSNR values of the output images of Recurrent-MZ with uniformly spaced, and non-uniformly spaced input images are calculated with respect to the ground truth, corresponding image. Blue: Outputs of Recurrent-MZ (M=3) for uniformly spaced inputs, Red: Outputs of Recurrent-MZ (M=3) for non-uniformly spaced inputs. Dashed lines indicate the axial positions of the input 2D images.

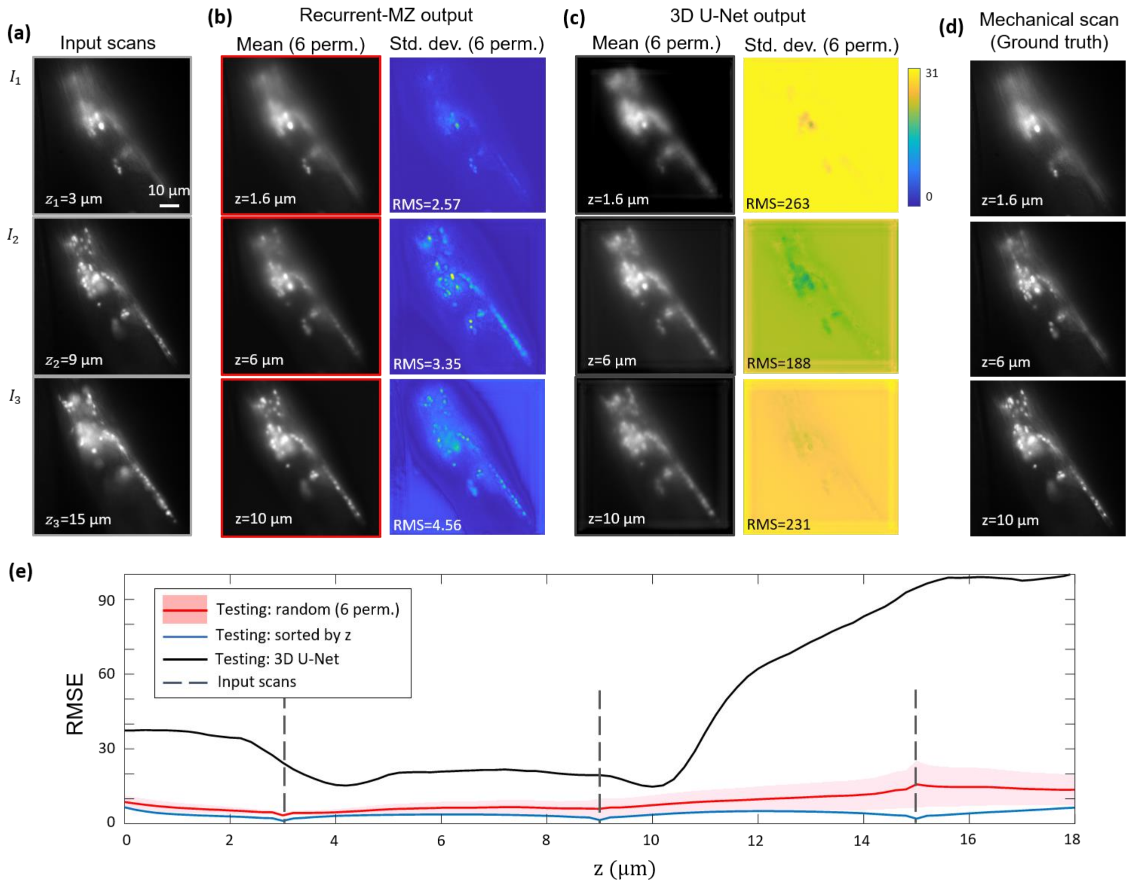

Fig. S5 Input image permutation invariance of Recurrent-MZ compared against the failure of 3D U-Net due to input image permutations. Recurrent-MZ (M=3) and 3D U-Net were trained with inputs sorted by z and blindly tested on new samples with 6 random permutations of the input images. (a) The input scans sorted by z, (b) the mean output and standard variance generated by Recurrent-MZ over 6 input image permutations, (c) the mean output and pixel-wise standard variance generated by 3D U-Net over 6 input image permutations, (d) the ground truth images obtained by mechanical scanning, (e) RMSE vs. z plot. Red solid line: average RMSE of the output images generated by Recurrent-MZ over 6 random permutation of the inputs;

Pink shadow: standard variance of the RMSE of the output images generated by Recurrent-MZ over 6 random permutation of the inputs; Blue solid line: RMSE of the output images generated by Recurrent-MZ with inputs sorted by *z*; Black solid line: average RMSE of the output images generated by 3D U-Net over 6 random permutation of the inputs.

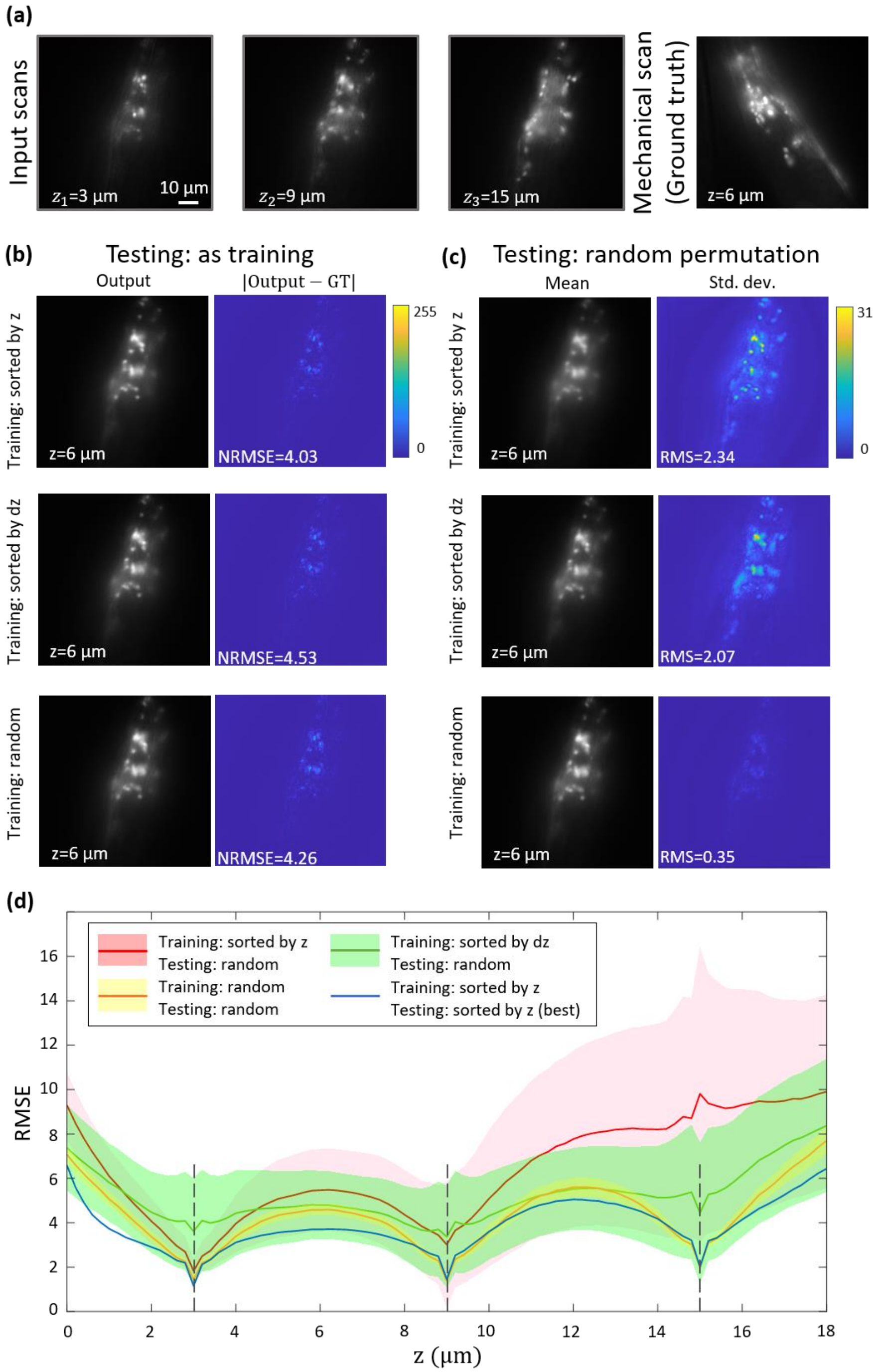

Fig. S6 Recurrent-MZ inference performance with different training schemes. (a) The input sequence and ground truth image of the test FOV.

Recurrent-MZ (M=3) was trained, separately, with input sequences sorted by z, sorted by dz, as well as randomly sorted images. The corresponding Recurrent-MZ networks were then tested with (b) the same image sorting used in training, and (c) 6 random permutations of the original input sequence. (d) The RMSE values of the output images of Recurrent-MZ trained using these three different schemes.

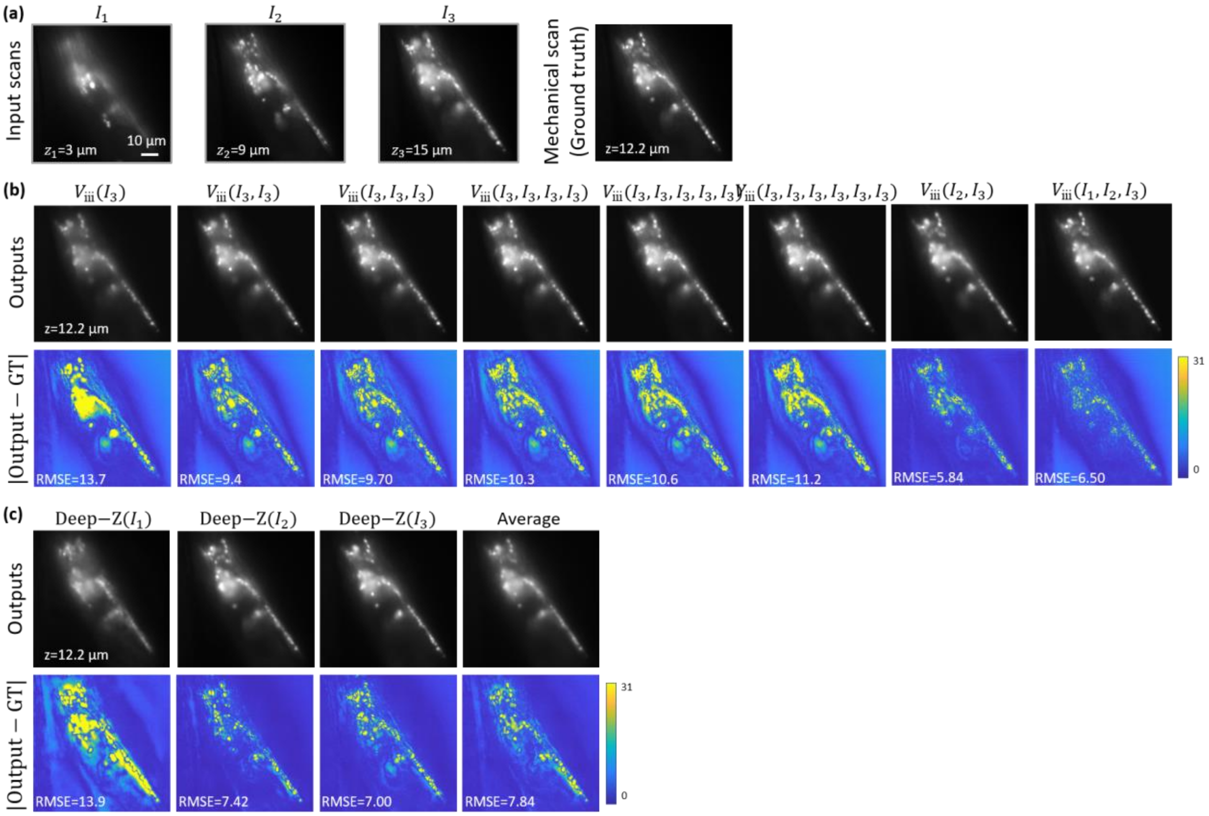

Fig. S7 Repetition invariance of Recurrent-MZ. Recurrent-MZ (M=3) was trained with input sequences with 3 input images (I_1, I_2 and I_3), but tested by repeatedly feeding the input image (I_3). (a) The input images/scans and the corresponding mechanical scan (ground truth) image. (b) Output images of

Recurrent-MZ (M=3) with the repetition of the nearest input (I_3), 2 nearest inputs (I_2, I_3) and all three input images. (c) The outputs of Deep-Z with single input (I_1, I_2 or I_3), and the pixel-wise average of three Deep-Z outputs, i.e., Deep-Z(I_1), Deep-Z(I_2) and Deep-Z(I_3). The range of grayscale images is 255 while that of standard variance images is 31.

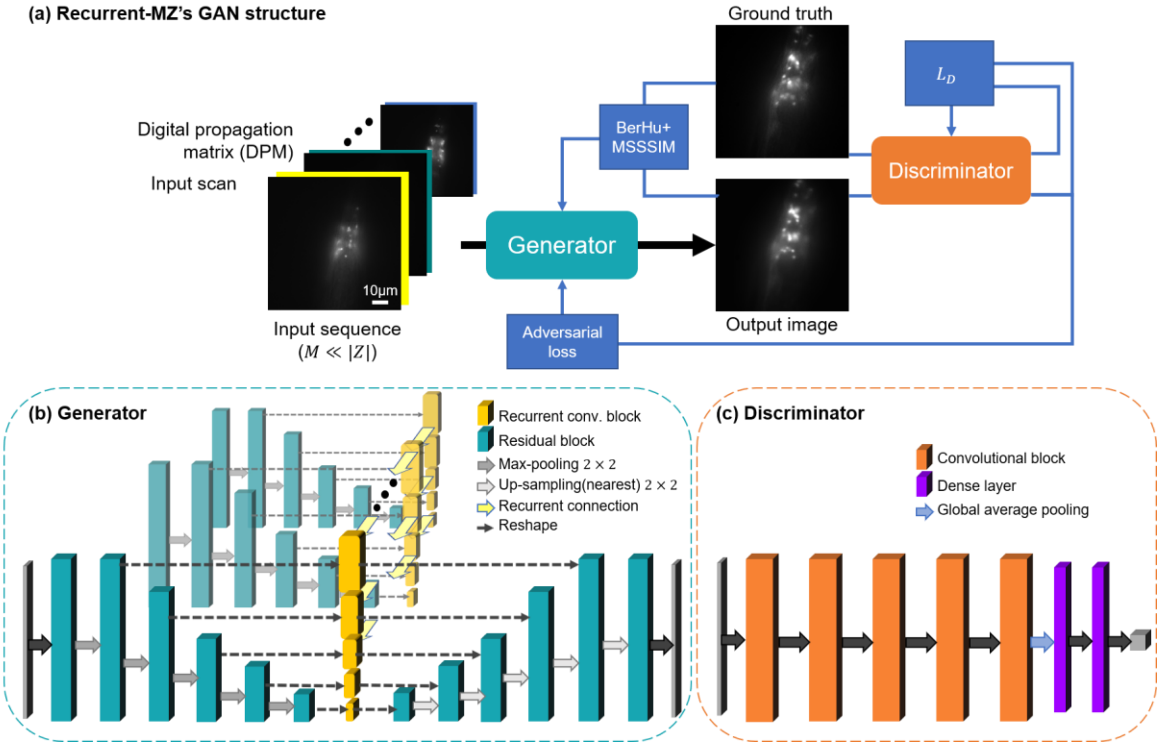

Fig. S8 Detailed network structure of Recurrent-MZ. (a) The GAN structure and the data flow of Recurrent-MZ. (b) The generator structure of Recurrent-MZ. (c) The discriminator structure used for training Recurrent-MZ.

Video S1. Volumetric imaging of *C. elegans* using Recurrent-MZ. Recurrent-MZ takes in the 2 nearest input scans (M=2) to each output plane using input images acquired at $z =$

$3, 9,$ and $15\ \mu\text{m}$. The reconstructed volume ranges from $z = 0\ \mu\text{m}$ to $z = 18\ \mu\text{m}$. Scale bar: $10\ \mu\text{m}$.

Video S2. Volumetric imaging of fluorescence nanobeads using Recurrent-MZ. Recurrent-MZ takes in 3 input images (*M*=3) of the 50nm fluorescence nanobead-sample imaged at $z = 3, 6,$ and $9\ \mu\text{m}$. The reconstructed volume ranges from $z = 0\ \mu\text{m}$ to $z = 10\ \mu\text{m}$. Scale bar: $10\ \mu\text{m}$.

Video S3. Cross-modality volumetric imaging of *C. elegans* using Recurrent-MZ+. Recurrent-MZ+ (*M*=3) propagates 3 wide-field input images into a 3D image stack, matching confocal microscopy images of the same sample. Scale bar: 10 μm.

References

1 Barbastathis G., Ozcan A. & Situ G. On the use of deep learning for computational imaging. *Optica* **6**, 921-943 (2019).

2 Kress R. Numerical Analysis. (New York: Springer, 1998).

3 Akima H. A New Method of Interpolation and Smooth Curve Fitting Based on Local Procedures. *Journal of the ACM* **17**, 589–602 (1970).

4 Akima H. A method of bivariate interpolation and smooth surface fitting based on local procedures. *Communications of the ACM* **17**, 18–20 (1974).
